# (2-Benzoyl­phen­yl)(3,4-dimethyl­phen­yl)methanone

**DOI:** 10.1107/S1600536811029965

**Published:** 2011-07-30

**Authors:** G. Jagadeesan, K. Sethusankar, R. Sivasakthikumaran, Arasambattu K. Mohanakrishnan

**Affiliations:** a Department of Physics, Dr MGR Educational and Research Institute University, Chennai 600 095, India; b Department of Physics, RKM Vivekananda College (Autonomous), Chennai 600 004, India; c Department of Organic Chemistry, University of Madras, Maraimalai Campus, Chennai 600 025, India

## Abstract

In the title compound, C_22_H_18_O_2_, the central benzene ring forms dihedral angles of 76.0 (1) and 73.1 (1)° with the phenyl ring and dimethyl-substituted benzene ring, respectively. The carbonyl-group O atoms deviate significantly from the phenyl ring and the dimethyl-substituted benzene ring [−0.582 (12) and 0.546 (12) Å, respectively]. The crystal packing is stabilized by C—H⋯π inter­actions.

## Related literature

For the synthesis of heterocyclic compounds, see: Hirsch & Bailey (1978 ▶). For chelating reagents of metallic systems, see: Liang *et al.* (2003 ▶). For related bond-length and angle values, see: Judaš & Kaitner (2005) ▶. For related structures, see: Khan *et al.* (2009 ▶); Narayanan *et al.* (2011 ▶).
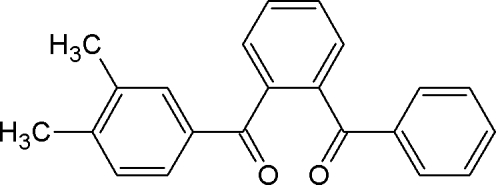


## Experimental

### 

#### Crystal data

C_22_H_18_O_2_
*M* *_r_* = 314.36
Monoclinic, 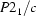

*a* = 17.8606 (14) Å
*b* = 7.7590 (6) Å
*c* = 11.9722 (11) Å
β = 93.942 (3)°
*V* = 1655.2 (2) Å^3^
*Z* = 4
Mo *K*α radiation
μ = 0.08 mm^−1^
*T* = 295 K
0.30 × 0.25 × 0.20 mm

#### Data collection

Bruker Kappa APEXII CCD diffractometer
17270 measured reflections
3862 independent reflections
2827 reflections with *I* > 2σ(*I*)
*R*_int_ = 0.030

#### Refinement

*R*[*F* ^2^ > 2σ(*F* ^2^)] = 0.044
*wR*(*F* ^2^) = 0.129
*S* = 1.01
3862 reflections
219 parameters
H-atom parameters constrained
Δρ_max_ = 0.22 e Å^−3^
Δρ_min_ = −0.18 e Å^−3^

### 

Data collection: *APEX2* (Bruker, 2008 ▶); cell refinement: *SAINT* (Bruker, 2008 ▶); data reduction: *SAINT*; program(s) used to solve structure: *SHELXS97* (Sheldrick, 2008 ▶); program(s) used to refine structure: *SHELXL97* (Sheldrick, 2008 ▶); molecular graphics: *ORTEP-3* (Farrugia, 1997 ▶); software used to prepare material for publication: *SHELXL97* and *PLATON* (Spek, 2009 ▶).

## Supplementary Material

Crystal structure: contains datablock(s) I, global. DOI: 10.1107/S1600536811029965/rk2286sup1.cif

Structure factors: contains datablock(s) I. DOI: 10.1107/S1600536811029965/rk2286Isup2.hkl

Supplementary material file. DOI: 10.1107/S1600536811029965/rk2286Isup3.cml

Additional supplementary materials:  crystallographic information; 3D view; checkCIF report

## Figures and Tables

**Table 1:** Hydrogen-bond geometry (Å, °) *Cg*1 is the centroid of the C1–C6 ring and *Cg*3 is the centroid of the C15–C20 ring.

*D*—H⋯*A*	*D*—H	H⋯*A*	*D*⋯*A*	*D*—H⋯*A*
C4—H4⋯*Cg*1^i^	0.93	2.86	3.747 (2)	159
C21—H21*A*⋯*Cg*3^ii^	0.96	2.91	3.8144 (17)	157
C22—H22*C*⋯*Cg*3^iii^	0.96	2.78	3.6484 (17)	152

Symmetry codes: (i) 
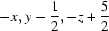
; (ii) 
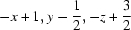
; (iii) 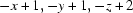.

**Fig. 1.**
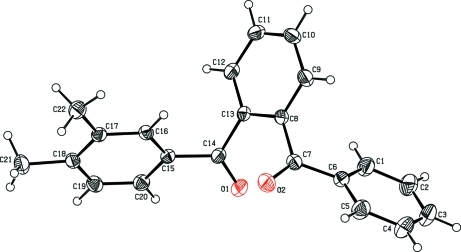
The molecular structure of the title compound with the atom numbering scheme. Displacement ellipsoids are drawn at the 30% probability level. H atoms are presented as a small spheres of arbitrary radius.

## supplementary crystallographic information

### Comment

Diketones are important synthetic intermediates and starting materials in the synthesis of many heterocyclic compounds (Hirsch & Bailey, 1978) and also employed as effective chelating reagents for a large number of metallic systems (Liang *et al.*, 2003).

X-ray analysis confirms the molecular structure and atom connectivity of the title compound as illustrated in the Fig. 1. The bond lengths and bond angles are normal and correspond to those observed in 2-benzyl-1,3-diphenylpropane-1,3-dione (Judaš & Kaitner, 2005). The central phenyl ring (C8–C13) of the compound forms dihedral angles of 76.0 (1)° and 73.1 (1)° with the other phenyl rings (C1–C6) and (C15–C20), respectively. The central phenyl ring (C8–C13) forms dihedral angles of 53.3 (6)° and 58.8 (6)° with the mean plane of the ketone groups (C13–C15/O1) and (C6–C8/O2), respectively.

In the dimethyl substituted phenyl ring (C15–C20) the deviation of atoms C21 and C22 are -0.014 (2) Å and -0.049 (2) Å, respectively. The atom O1 deviates by -0.582 (1) Å from the plane of the phenyl ring (C1–C6). Also the atom O2 deviate by 0.546 (1) Å from the plane of the phenyl ring (C15–C20). The title compound exhibits the structural similarities with the already reported related structures (Khan *et al.*, 2009; Narayanan *et al.*, 2011).

The molecular structure is stabilized by C—H···*Cg* interactions - look Table 1. The *Cg*1 is center of gravity of (C1–C6) ring and *Cg*3 is center of gravity of (C15–C20) ring. Symmetry codes: (i) -*x*, *y* - 1/2, -*z* + 5/2; (ii) -*x* + 1, *y* - 1/2, -*z* + 3/2; (iii) -*x* + 1, -*y* + 1, -*z* + 2.

### Experimental

To a stirred suspension of 1-(3,4-dimethylphenyl)-3-phenyl-2-benzofuran (1 g, 3.22 mmol) in dry THF (20 ml), lead tetraacetate (1.52 g, 3.42 mmol) was added and refluxed at 343 K for half an hour. The reaction mixture was then poured into water (200 ml) and extracted with ethyl acetate (2 × 20 ml), washed with brine solution and dried (Na_2_SO_4_). The removal of solvent *in vacuo* afforded crude product. The crude product upon crystallization from methanol furnished the title compound as a colourless solid.

### Refinement

Hydrogen atoms were placed in calculated positions with C—H = 0.93 Å and 0.96 Å refined in the riding model with fixed isotropic displacement parameters: *U*_iso_(H) = 1.5*U*_eq_(C) for methyl atoms and *U*_iso_(H) = 1.2*U*_eq_(C) for aryl atoms.

### Crystal data

**Table:**

C_22_H_18_O_2_	*F*(000) = 664
*M**_r_* = 314.36	*D*_x_ = 1.262 Mg m^−^^3^
Monoclinic, *P*2_1_/*c*	Mo *K*α radiation, λ = 0.71073 Å
Hall symbol: -P 2ybc	Cell parameters from 3862 reflections
*a* = 17.8606 (14) Å	θ = 2.3–27.7°
*b* = 7.7590 (6) Å	µ = 0.08 mm^−^^1^
*c* = 11.9722 (11) Å	*T* = 295 K
β = 93.942 (3)°	Block, colourless
*V* = 1655.2 (2) Å^3^	0.30 × 0.25 × 0.20 mm
*Z* = 4	

### Data collection

**Table:**

Bruker Kappa APEXII CCD diffractometer	2827 reflections with *I* > 2σ(*I*)
Radiation source: fine-focus sealed tube	*R*_int_ = 0.030
graphite	θ_max_ = 27.7°, θ_min_ = 2.3°
ω scans	*h* = −23→21
17270 measured reflections	*k* = −10→10
3862 independent reflections	*l* = −15→15

### Refinement

**Table:**

Refinement on *F*^2^	Primary atom site location: structure-invariant direct methods
Least-squares matrix: full	Secondary atom site location: difference Fourier map
*R*[*F*^2^ > 2σ(*F*^2^)] = 0.044	Hydrogen site location: inferred from neighbouring sites
*wR*(*F*^2^) = 0.129	H-atom parameters constrained
*S* = 1.01	*w* = 1/[σ^2^(*F*_o_^2^) + (0.0581*P*)^2^ + 0.4044*P*] where *P* = (*F*_o_^2^ + 2*F*_c_^2^)/3
3862 reflections	(Δ/σ)_max_ < 0.001
219 parameters	Δρ_max_ = 0.22 e Å^−^^3^
0 restraints	Δρ_min_ = −0.18 e Å^−^^3^

### Special details

**Table:**

Geometry. All s.u.'s (except the s.u. in the dihedral angle between two l.s. planes) are estimated using the full covariance matrix. The cell s.u.'s are taken into account individually in the estimation of s.u.'s in distances, angles and torsion angles; correlations between s.u.'s in cell parameters are only used when they are defined by crystal symmetry. An approximate (isotropic) treatment of cell s.u.'s is used for estimating s.u.'s involving l.s. planes.
Refinement. Refinement of *F*^2^ against ALL reflections. The weighted *R*-factor *wR* and goodness of fit *S* are based on *F*^2^, conventional *R*-factors *R* are based on *F*, with *F* set to zero for negative *F*^2^. The threshold expression of *F*^2^ > σ(*F*^2^) is used only for calculating *R*-factors(gt) *etc*. and is not relevant to the choice of reflections for refinement. *R*-factors based on *F*^2^ are statistically about twice as large as those based on *F*, and *R*-factors based on ALL data will be even larger.

### Fractional atomic coordinates and isotropic or equivalent isotropic displacement parameters (Å^2^)

**Table:**

	*x*	*y*	*z*	*U*_iso_*/*U*_eq_	
C1	0.05252 (9)	0.7620 (2)	1.04279 (14)	0.0501 (4)	
H1	0.0669	0.8384	0.9884	0.060*	
C2	−0.02238 (9)	0.7235 (3)	1.04997 (17)	0.0655 (5)	
H2	−0.0582	0.7729	0.9998	0.079*	
C3	−0.04421 (11)	0.6128 (3)	1.1308 (2)	0.0731 (6)	
H3	−0.0948	0.5871	1.1354	0.088*	
C4	0.00862 (12)	0.5398 (3)	1.20492 (18)	0.0722 (6)	
H4	−0.0064	0.4659	1.2603	0.087*	
C5	0.08396 (10)	0.5754 (2)	1.19788 (14)	0.0555 (4)	
H5	0.1196	0.5245	1.2477	0.067*	
C6	0.10627 (8)	0.68748 (18)	1.11613 (12)	0.0397 (3)	
C7	0.18732 (8)	0.71909 (18)	1.10391 (11)	0.0375 (3)	
C8	0.20890 (7)	0.88500 (17)	1.05002 (12)	0.0361 (3)	
C9	0.19085 (8)	1.04058 (19)	1.09856 (14)	0.0481 (4)	
H9	0.1628	1.0414	1.1612	0.058*	
C10	0.21426 (9)	1.1940 (2)	1.05444 (17)	0.0579 (5)	
H10	0.2032	1.2977	1.0887	0.070*	
C11	0.25395 (9)	1.1945 (2)	0.95989 (18)	0.0607 (5)	
H11	0.2678	1.2984	0.9285	0.073*	
C12	0.27318 (8)	1.04049 (19)	0.91177 (15)	0.0516 (4)	
H12	0.3010	1.0412	0.8489	0.062*	
C13	0.25147 (7)	0.88492 (17)	0.95623 (12)	0.0377 (3)	
C14	0.26870 (7)	0.71987 (17)	0.89837 (11)	0.0364 (3)	
C15	0.34810 (7)	0.68460 (16)	0.87551 (11)	0.0338 (3)	
C16	0.40683 (7)	0.74660 (17)	0.94594 (11)	0.0343 (3)	
H16	0.3961	0.8163	1.0060	0.041*	
C17	0.48140 (7)	0.70753 (16)	0.92943 (11)	0.0343 (3)	
C18	0.49709 (8)	0.60617 (17)	0.83744 (11)	0.0363 (3)	
C19	0.43783 (8)	0.54581 (18)	0.76678 (11)	0.0412 (3)	
H19	0.4482	0.4791	0.7052	0.049*	
C20	0.36435 (8)	0.58169 (18)	0.78516 (11)	0.0395 (3)	
H20	0.3257	0.5375	0.7375	0.047*	
C21	0.57643 (9)	0.5594 (2)	0.81499 (14)	0.0500 (4)	
H21A	0.5760	0.4796	0.7537	0.075*	
H21B	0.6007	0.5073	0.8806	0.075*	
H21C	0.6032	0.6615	0.7964	0.075*	
C22	0.54257 (9)	0.7725 (2)	1.00999 (13)	0.0472 (4)	
H22A	0.5211	0.8371	1.0682	0.071*	
H22B	0.5756	0.8454	0.9712	0.071*	
H22C	0.5703	0.6767	1.0423	0.071*	
O1	0.21870 (6)	0.61966 (14)	0.87004 (10)	0.0530 (3)	
O2	0.23523 (6)	0.61759 (14)	1.13704 (10)	0.0545 (3)	

### Atomic displacement parameters (Å^2^)

**Table:**

	*U*^11^	*U*^22^	*U*^33^	*U*^12^	*U*^13^	*U*^23^
C1	0.0392 (8)	0.0533 (9)	0.0576 (9)	−0.0033 (7)	0.0023 (7)	0.0058 (7)
C2	0.0382 (9)	0.0726 (12)	0.0846 (13)	−0.0041 (8)	−0.0035 (9)	0.0008 (10)
C3	0.0435 (10)	0.0785 (14)	0.0991 (15)	−0.0173 (9)	0.0185 (10)	−0.0055 (12)
C4	0.0656 (13)	0.0712 (13)	0.0830 (13)	−0.0172 (10)	0.0288 (11)	0.0111 (11)
C5	0.0547 (10)	0.0568 (10)	0.0560 (9)	−0.0037 (8)	0.0110 (8)	0.0100 (8)
C6	0.0370 (7)	0.0390 (7)	0.0436 (7)	−0.0022 (6)	0.0066 (6)	−0.0025 (6)
C7	0.0367 (7)	0.0368 (7)	0.0390 (7)	0.0029 (6)	0.0020 (6)	−0.0020 (6)
C8	0.0263 (6)	0.0331 (7)	0.0485 (8)	0.0009 (5)	−0.0004 (6)	−0.0019 (6)
C9	0.0377 (8)	0.0434 (8)	0.0630 (9)	0.0063 (6)	0.0033 (7)	−0.0088 (7)
C10	0.0392 (8)	0.0334 (8)	0.1004 (14)	0.0050 (7)	−0.0008 (9)	−0.0130 (8)
C11	0.0389 (8)	0.0320 (8)	0.1117 (15)	−0.0032 (7)	0.0077 (9)	0.0111 (9)
C12	0.0362 (8)	0.0408 (8)	0.0791 (11)	−0.0023 (6)	0.0132 (8)	0.0099 (8)
C13	0.0254 (6)	0.0340 (7)	0.0537 (8)	−0.0008 (5)	0.0035 (6)	0.0017 (6)
C14	0.0319 (7)	0.0358 (7)	0.0419 (7)	−0.0028 (6)	0.0048 (6)	0.0031 (6)
C15	0.0323 (7)	0.0313 (6)	0.0382 (7)	−0.0011 (5)	0.0046 (5)	0.0030 (5)
C16	0.0369 (7)	0.0321 (7)	0.0347 (6)	0.0004 (5)	0.0074 (5)	−0.0017 (5)
C17	0.0343 (7)	0.0320 (6)	0.0365 (7)	−0.0009 (5)	0.0027 (5)	0.0047 (5)
C18	0.0370 (7)	0.0336 (7)	0.0394 (7)	0.0045 (6)	0.0091 (6)	0.0066 (5)
C19	0.0477 (8)	0.0401 (8)	0.0366 (7)	0.0035 (6)	0.0080 (6)	−0.0057 (6)
C20	0.0397 (8)	0.0404 (7)	0.0381 (7)	−0.0031 (6)	0.0009 (6)	−0.0031 (6)
C21	0.0418 (8)	0.0538 (9)	0.0557 (9)	0.0106 (7)	0.0122 (7)	0.0022 (7)
C22	0.0397 (8)	0.0523 (9)	0.0489 (8)	−0.0027 (7)	−0.0023 (6)	−0.0010 (7)
O1	0.0388 (6)	0.0506 (7)	0.0701 (7)	−0.0122 (5)	0.0088 (5)	−0.0117 (5)
O2	0.0459 (6)	0.0471 (6)	0.0703 (7)	0.0103 (5)	0.0029 (5)	0.0112 (5)

### Geometric parameters (Å, °)

**Table:**

C1—C2	1.379 (2)	C12—C13	1.3853 (19)
C1—C6	1.382 (2)	C12—H12	0.9300
C1—H1	0.9300	C13—C14	1.4980 (19)
C2—C3	1.371 (3)	C14—O1	1.2146 (16)
C2—H2	0.9300	C14—C15	1.4880 (18)
C3—C4	1.372 (3)	C15—C16	1.3861 (19)
C3—H3	0.9300	C15—C20	1.3911 (18)
C4—C5	1.382 (3)	C16—C17	1.3932 (18)
C4—H4	0.9300	C16—H16	0.9300
C5—C6	1.388 (2)	C17—C18	1.3973 (18)
C5—H5	0.9300	C17—C22	1.494 (2)
C6—C7	1.4854 (19)	C18—C19	1.390 (2)
C7—O2	1.2095 (17)	C18—C21	1.5046 (19)
C7—C8	1.5020 (19)	C19—C20	1.374 (2)
C8—C9	1.3873 (19)	C19—H19	0.9300
C8—C13	1.399 (2)	C20—H20	0.9300
C9—C10	1.379 (2)	C21—H21A	0.9600
C9—H9	0.9300	C21—H21B	0.9600
C10—C11	1.376 (3)	C21—H21C	0.9600
C10—H10	0.9300	C22—H22A	0.9600
C11—C12	1.380 (2)	C22—H22B	0.9600
C11—H11	0.9300	C22—H22C	0.9600
			
C2—C1—C6	120.27 (16)	C12—C13—C8	119.34 (13)
C2—C1—H1	119.9	C12—C13—C14	119.69 (13)
C6—C1—H1	119.9	C8—C13—C14	120.79 (12)
C3—C2—C1	120.25 (18)	O1—C14—C15	121.51 (12)
C3—C2—H2	119.9	O1—C14—C13	120.39 (12)
C1—C2—H2	119.9	C15—C14—C13	118.09 (11)
C2—C3—C4	119.92 (17)	C16—C15—C20	118.85 (12)
C2—C3—H3	120.0	C16—C15—C14	121.02 (12)
C4—C3—H3	120.0	C20—C15—C14	120.07 (12)
C3—C4—C5	120.49 (18)	C15—C16—C17	121.94 (12)
C3—C4—H4	119.8	C15—C16—H16	119.0
C5—C4—H4	119.8	C17—C16—H16	119.0
C4—C5—C6	119.76 (17)	C16—C17—C18	118.67 (12)
C4—C5—H5	120.1	C16—C17—C22	119.91 (12)
C6—C5—H5	120.1	C18—C17—C22	121.41 (13)
C1—C6—C5	119.30 (14)	C19—C18—C17	118.93 (12)
C1—C6—C7	120.48 (13)	C19—C18—C21	119.81 (13)
C5—C6—C7	120.13 (14)	C17—C18—C21	121.26 (13)
O2—C7—C6	122.17 (13)	C20—C19—C18	122.01 (13)
O2—C7—C8	120.16 (12)	C20—C19—H19	119.0
C6—C7—C8	117.67 (12)	C18—C19—H19	119.0
C9—C8—C13	119.44 (13)	C19—C20—C15	119.57 (13)
C9—C8—C7	119.47 (13)	C19—C20—H20	120.2
C13—C8—C7	120.94 (11)	C15—C20—H20	120.2
C10—C9—C8	120.37 (15)	C18—C21—H21A	109.5
C10—C9—H9	119.8	C18—C21—H21B	109.5
C8—C9—H9	119.8	H21A—C21—H21B	109.5
C11—C10—C9	120.26 (15)	C18—C21—H21C	109.5
C11—C10—H10	119.9	H21A—C21—H21C	109.5
C9—C10—H10	119.9	H21B—C21—H21C	109.5
C10—C11—C12	119.89 (15)	C17—C22—H22A	109.5
C10—C11—H11	120.1	C17—C22—H22B	109.5
C12—C11—H11	120.1	H22A—C22—H22B	109.5
C11—C12—C13	120.64 (15)	C17—C22—H22C	109.5
C11—C12—H12	119.7	H22A—C22—H22C	109.5
C13—C12—H12	119.7	H22B—C22—H22C	109.5
			
C6—C1—C2—C3	0.8 (3)	C7—C8—C13—C12	−177.13 (13)
C1—C2—C3—C4	0.0 (3)	C9—C8—C13—C14	−176.94 (13)
C2—C3—C4—C5	−0.8 (3)	C7—C8—C13—C14	7.6 (2)
C3—C4—C5—C6	0.8 (3)	C12—C13—C14—O1	−124.06 (16)
C2—C1—C6—C5	−0.8 (2)	C8—C13—C14—O1	51.2 (2)
C2—C1—C6—C7	175.82 (15)	C12—C13—C14—C15	55.03 (18)
C4—C5—C6—C1	0.0 (3)	C8—C13—C14—C15	−129.72 (14)
C4—C5—C6—C7	−176.65 (16)	O1—C14—C15—C16	−150.17 (14)
C1—C6—C7—O2	−153.93 (15)	C13—C14—C15—C16	30.75 (18)
C5—C6—C7—O2	22.7 (2)	O1—C14—C15—C20	27.0 (2)
C1—C6—C7—C8	27.09 (19)	C13—C14—C15—C20	−152.12 (13)
C5—C6—C7—C8	−156.27 (14)	C20—C15—C16—C17	−0.6 (2)
O2—C7—C8—C9	−118.70 (16)	C14—C15—C16—C17	176.55 (12)
C6—C7—C8—C9	60.30 (17)	C15—C16—C17—C18	1.55 (19)
O2—C7—C8—C13	56.75 (19)	C15—C16—C17—C22	−177.87 (13)
C6—C7—C8—C13	−124.25 (14)	C16—C17—C18—C19	−0.98 (19)
C13—C8—C9—C10	0.4 (2)	C22—C17—C18—C19	178.44 (13)
C7—C8—C9—C10	175.91 (14)	C16—C17—C18—C21	−179.98 (12)
C8—C9—C10—C11	1.8 (2)	C22—C17—C18—C21	−0.6 (2)
C9—C10—C11—C12	−2.8 (3)	C17—C18—C19—C20	−0.5 (2)
C10—C11—C12—C13	1.5 (3)	C21—C18—C19—C20	178.50 (13)
C11—C12—C13—C8	0.8 (2)	C18—C19—C20—C15	1.5 (2)
C11—C12—C13—C14	176.08 (15)	C16—C15—C20—C19	−0.9 (2)
C9—C8—C13—C12	−1.7 (2)	C14—C15—C20—C19	−178.09 (12)

### Hydrogen-bond geometry (Å, °)

**Table:**

*Cg*1 is the centroid of the C1–C6 ring and *Cg*3 is the centroid of the C15–C20 ring.

**Table:**

*D*—H···*A*	*D*—H	H···*A*	*D*···*A*	*D*—H···*A*
C4—H4···Cg1^i^	0.93	2.86	3.747 (2)	159
C21—H21A···Cg3^ii^	0.96	2.91	3.8144 (17)	157
C22—H22C···Cg3^iii^	0.96	2.78	3.6484 (17)	152

Symmetry codes: (i) −*x*, *y*−1/2, −*z*+5/2; (ii) −*x*+1, *y*−1/2, −*z*+3/2; (iii) −*x*+1, −*y*+1, −*z*+2.
